# Body-size evolution in gastropods across the Plio-Pleistocene extinction in the western Atlantic

**DOI:** 10.1371/journal.pone.0313060

**Published:** 2024-12-13

**Authors:** Brendan M. Anderson, Elizabeth Petsios, Jessica Behn, Amy Betz, Warren D. Allmon, Bruce S. Lieberman, Jonathan R. Hendricks

**Affiliations:** 1 Paleontological Research Institution, Ithaca, NY, United States of America; 2 Department of Geosciences, Baylor University, Waco, TX, United States of America; 3 Biological Sciences Program, Cornell University, Ithaca, NY, United States of America; 4 Biodiversity Institute and Department of Ecology & Evolutionary Biology, Dyche Hall, University of Kansas, Lawrence, KS, United States of America; 5 Milwaukee Public Museum, Milwaukee, WI, United States of America; University of Basrah, IRAQ

## Abstract

The Plio-Pleistocene turnover event in the western Atlantic following the closure of the Central American Seaway involved high rates of extinction for both gastropod and bivalve molluscs. This extinction was associated with declining nutrient conditions and has been presumed to be associated with a decrease in molluscan body size. Previous work which has been concordant with this expectation, however, has either focused on bivalves or not considered the effects of the recovery post extinction. In three phylogenetically diverse clades, we found that body-size evolution in gastropods across the turnover event is likely tied to ecology. One clade increased in size, one decreased, and another exhibited no substantial change. Individual species lineages exhibit a mixture of microevolutionary changes from the Pliocene to today. This study indicates that gastropod body-size evolution may be more complex than in bivalves, with ecology and other functional traits playing a significant role. Macroevolutionary processes, especially whether a clade re-radiated post extinction, were found to be important. Indeed, a low portion of extant diversity consists of survivors from clades that increased in size or have similar size distributions among their species relative to the Pliocene.

## Introduction

### The western Atlantic Plio-Pleistocene extinctions

Major environmental change and high faunal turnover have made the Neogene–recent western Atlantic a model system for studying the macroevolutionary dynamics that led to the region’s modern coastal ecosystems [1,2]. This system is characterized not only by high extinction, peaking around 2 Ma, but also with significant origination leading to similar modern levels of diversity relative to the Plio-Pleistocene, albeit with different taxonomic composition and differences in species’ abundance [2–6]. The extinction was regional but severe and basin wide, with around 70% of gastropod species [3,7], and 47–65% of bivalves [3,8] going extinct. This system is well documented, with a rich fossil record, and the extinction occurred in relatively recent geologic history, making it ideal for studying not only how extinction impacts ecologically relevant traits but also how recovery can interact with apparently selective extinction pulses. Most work studying this event has focused on the southwest Caribbean, especially Panama [2,6,9,10], though the event is known to have impacted Florida as well [3,7,11,12].

The turnover event was not simply a transient perturbation but rather represented a permanent shift in ecosystems associated with changing environmental conditions. Nutrients declined in the western Atlantic following the closure of the Central American Seaway. The formation of the Central American Isthmus was completed ~2.8 Ma [13,14], cutting off connections to eastern Pacific waters. Resulting oceanographic changes impacted local upwelling [2,12,15–17]. Subsequent global cooling during the Pleistocene modified the hydrologic cycle and may have impacted delivery of nutrients via runoff [18,19]. Hardground habitats and seagrasses also expanded, altering the habitats occupied by marine mollusks [2,5,6,20]. Some studies indicate that predation intensity may also have declined in the region but evidence for changes to predation intensity is limited [21–23]. A possible increase in parasitism in some taxa [24] may be related to declines in predation intensity [25,26], but it is difficult to determine how much of this signal may be taphonomic without further study. In contrast, Sime & Kelley [27] showed stability in predation intensity across the turnover event and highlighted the potential importance for regional variation in response.

Habitat association [2,6], feeding mode [2,6,14,28], and larval ecology [2,17,29,30] have each been observed to be associated with survivorship in this event and success across the boundary, as has metabolic rate, a trait related to body size [1].

### Body-size evolution

The impact of evolutionary processes and mass extinction on macroinvertebrate body size has long been of paleontological interest (e.g., 31–35). From a theoretical perspective, selection pressures can act in the same or opposite directions on species within a clade and the constituent populations of each species [36–41]. This results in complex relationships between body-size variation across species-rich clades and among organisms within individual species. Body-size evolution is also an interesting feature to examine in the context of the Plio-Pleistocene of the western Atlantic (PPWA) because the relationship between available nutrients and the distribution of average or maximum species body sizes in a community may not be tied to the microevolutionary responses observed in the constituent species under high and low nutrient conditions. For example, the distribution of sizes in a population may be centered around a smaller mean under low-nutrient conditions. Under high-nutrient conditions, however, it is possible the community supports more species overall, including both small and large species.

Marine mollusks represent a major component of the Mesozoic to Pleistocene fossil record. Compared with other marine poikilotherms, mollusks exhibit distinctive body-size trends and responses to environmental changes [42] including the effect of body size on species duration and extinction risk (e.g., work by Payne & Heim [34], Monarrez et al. [35], Pietsch et al. [41], Jablonski [43], and Crampton et al. [44]). Some studies have recovered conflicting trends across different clades [44,45] whereas others have revealed no trend [43]. There can even be conflicting trends in body-size responses to different extinction events [46]. This suggests that understanding body-size evolution in different clades of mollusks is a complex problem affected by several factors. In some cases, the most pronounced factors are how body size influences group-level characters like species’ geographic range (see Jablonski & Hunt [47] for discussion of geographic range size as a group-level character), especially via the relationship between geographic range size and extinction risk. In other cases, body size is correlated with other organismal traits such as fecundity or other aspects of an organism’s life history or ecology (see reference 41 for further discussion of traits that have been linked to body size in a variety of taxa), and these other factors may be what is primarily determining extinction risk. Lastly, there are factors related to chance and contingency [48].

Recently, Monarrez et al. [49], in an important paper, demonstrated that geographic range is likely to be a more significant influence on survivorship than body size. However, selectivity for body size was distinguishable from selectivity on geographic range in their analyses. Further, extinction models that included body size were preferred over geographic range-only models for gastropods but not bivalves [49], indicating patterns may vary across mollusks. Smaller-bodied gastropod genera were found to have higher extinction risk during both background and mass extinction events, whereas bivalves exhibited almost no selectivity on body size during mass extinctions [49].

While global-scale patterns of selectivity during mass and background extinctions observed at the generic level may yield consistent long-term patterns, the regional event in the PPWA may not conform to this pattern. Indeed, Monarrez et al. [49] noted that local extinction dynamics may deviate from the broader-scale patterns they observed and additively contribute to extinction dynamics during background intervals. The extinction event in the PPWA has been characterized previously as resulting in a smaller body sized post-extinction fauna [5].

In the PPWA, chionine bivalves (placed in *Chione*, *Chionopsis*, and *Lirophora*) decreased in size in the western Atlantic across the boundary [50]. Corbulid bivalves also exhibited a body-size decline for the PPWA, yet over the same interval in the eastern Pacific body size increased [51,52]. *Strombina*-group gastropods did not exhibit a significant long-term trend in body-size change in the Neogene western Atlantic, although they did show a significant increase in size in the eastern Pacific [29]. However, it is notable that the three extant species in the western Atlantic are larger compared to Plio-Pleistocene forms [29].

The differences between patterns in the PPWA and other regions and times may be attributable to the very specific environmental changes experienced in the PPWA, such as changes in nutrient availability. The association between the PPWA extinction event and a shift to lower-nutrient conditions could be expected to influence the relationship between body size and extinction. For instance, body size in mollusks is generally correlated with nutrient availability [53], which declined in the post-Pliocene of the western Atlantic. Therefore, all other factors being equal, one may expect the pre-extinction fauna to consist of larger body-sized mollusks than the post-extinction fauna [54–58]. In this study, we tested the hypothesis that modern members of gastropod clades in the region differ in body size from the representatives of those same clades in PPWA. We compared all constituent species belonging to three gastropod clades from before and after the extinction, as well as examined body-size evolution in surviving species lineages across the extinction event.

## Geologic setting of the extinction

Fossiliferous units of the PPWA include primarily unlithified sands, silts, and clays, while the fossil record in Florida includes both sands and siliciclastic bearing carbonates (“shell beds”) [59–62]. The stratigraphic correlations among units of the United States Atlantic Coastal Plain and Florida are complex, with the chronostratigraphy of various units continuing to improve even in recent years [27,59,60,63–65]. Herein, we follow the stratigraphic framework used in Friend et al. [66] (S1 Fig), which was based on the work of Lyons [62], Saupe et al. [63], Campbell [67], Ward & Gilinsky [68], Allmon et al. [3], Hendricks [69], Kittle et al. [70], Dowsett et al. [65], and references therein.

**Fig 1:**
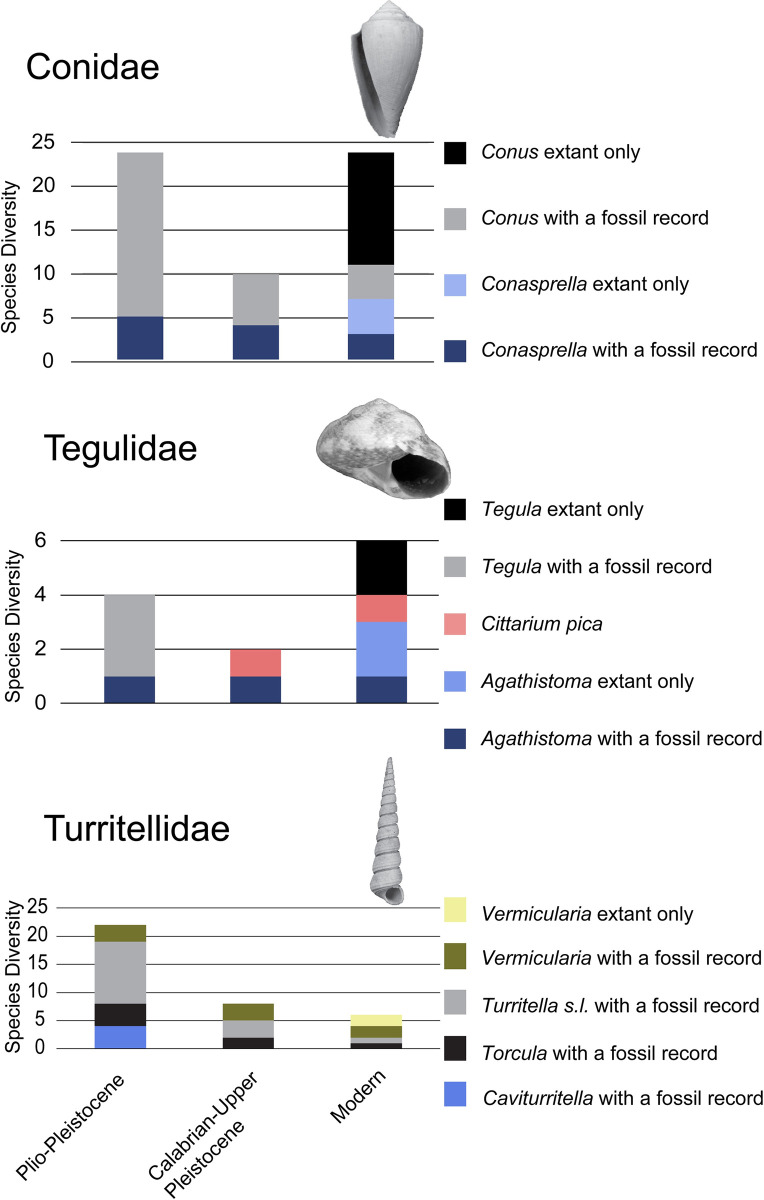
Diversity of each focal taxon in Florida and the Atlantic Coastal Plain throughout the study interval. Species are divided according to whether they have a fossil record or represent post-turnover origination. Plio-Pleistocene is Piacenzian–Gelasian, the next time bin is Calabrian–upper Pleistocene, and modern is present day diversity in the region. This time division was chosen rather than the end of the Pliocene because in Florida the Nashua Fm. and Pinecrest Beds within the Tamiami Fm. cross the Piacenzian-Gelasian boundary, and substantial faunal turnover occurred not at the Pliocene-Pleistocene boundary but was completed shortly thereafter. The interval designated Calabrian-Upper Pleistocene corresponds to the James City, Waccamaw, Bermont, Canepatch, and Ft. Thompson Formations (see S1 Fig). Our subsequent analyses do not depend on the selection of this post-extinction, but pre-modern time category, it is presented here to demonstrate the proportion of modern species that have fossil records dating to this time. *Torcula exoleta* (Linnaeus, 1758) is believed to be a direct descendant by anagenesis of *Torcula perattenuata* (Heilprin, 1886) [66] and is therefore represented as a lineage with a fossil record. Representative images of species are as follows: Conidae, *Conus adversarius* Conrad, 1840, University of Florida 66443, modified from Hendricks [69], plate 7, figure 13; Tegulidae, *Tegula exoluta* (Conrad, 1843) Paleontological Research Institution 70266, modified from the Neogene Atlas of Ancient life, used under Creative Commons Attribution-NonCommercial-ShareAlike 4.0 International License; Turritellidae, *Caviturritella magnasulcus* (Petuch, 1991) Carnegie Museum 35625, modified from Friend et al. [66], figure 16.

S1 **Fig** Stratigraphic correlation of important Pliocene-Pleistocene fossiliferous units of Florida and the Atlantic Coastal Plain. Modified from Friend et al. [66], text Fig 1.

The Piacenzian is represented in Virginia and North Carolina by the Yorktown Fm., in the Carolinas and Georgia by the Duplin and Raysor formations, and in Florida by portions of the Jackson Bluff, Nashua, and Tamiami formations (represented by Pinecrest beds 5–9 of the Tamiami Fm.). The Gelasian is represented by the Chowan River Fm. in Virginia and North Carolina and the Bear Bluff Fm. in North and South Carolina. In Florida, the upper portion of the Nashua and Tamiami formations (Pinecrest beds 2–4), and the Caloosahatchee Formation are considered Gelasian (earliest Pleistocene). The James City and Waccamaw formations overly the Chowan River and Bear Bluff formations in North and South Carolina, straddling the boundary between the Gelasian and subsequent Calabrian. The Canepatch Fm. in South Carolina and the Bermont and Ft. Thompson units in southern Florida appear to substantially post-date the extinction event. The Caloosahatchee, Bermont, and Ft. Thompson “formations” have been combined into the Okeechobee Fm. by Scott [71], but this name is not in wide use.Based on faunal differences these have been treated as separate units in the recent paleontological literature [22,66,72,73].

### Taxon selection

Three phylogenetically diverse families spanning the Plio-Pleistocene and recent were chosen for analysis and represent clades with distinct ecologies: Conidae (Caenogastropoda: Neogastropoda), Tegulidae (Vetigastropoda: Trochoidea), and non-*Vermicularia* Turritellidae (Caenogastropoda: Cerithioidea). These taxa were selected because each clade experienced notable turnover at the extinction, and each has at least one species lineage which survived from the Plio-Pleistocene to the modern (Fig 1; species list in S1 Table).

### Conidae

Cone snails are an extremely diverse family of venomous, predatory gastropods with high species diversity in both modern communities and fossil assemblages in Florida [69,74]. Multiple species frequently co-occur, most likely due to high degree of specialization on different prey species [75]. Conidae are also of evolutionary interest because of their extremely rapid rates of speciation [74,76]. Fossil Conidae of the western Atlantic Coastal Plain have also been subject to relatively recent systematic revision [69], which included the collection of measurements from several hundred fossil specimens. Twenty-four species of Conidae (19 of *Conus* and five of *Conasprella*) were present in the PPWA prior to the extinction interval. Twenty-four species also live in the region today (17 of *Conus* and seven of *Conasprella*), and eight of these species have a fossil record (three of *Conasprella* and five of *Conus*). *Conus patricius* was present in the PPWA, but is now restricted to the eastern Pacific. *Conus amphiurgus* has been reported from the Bermont Fm. [77], which we consider post-extinction, but we note that this has not been verified based on museum specimens [69].

### Tegulidae

Tegulids are grazers on micro- and macroalgae as well as sea grasses [78,79]. Recent species inhabit shallow water habitats with some species living within the splash zone and intertidal waters and others restricted to subtidal zones [80]. Florida fossil Tegulidae have been relatively understudied compared with the other families considered herein. Modern tegulids are represented in the region by two species of the genus *Agathistoma*, three of *Tegula*, and *Cittarium pica* (Linnaeus, 1758). There is some dispute as to whether *Cittarium pica*, which is significantly larger in body size than the other tegulid genera, is properly nested within Tegulidae or is a close relative [81], but we include it here and perform analyses both including and excluding this species. *Cittarium pica* appears in the late Pleistocene Miami Limestone (~130 ka based on uranium series dating [82,83]), which is post-extinction. One species of *Agathistoma* and three of *Tegula* were present in the PPWA. We treated *Tegula* (*Monodonta*) *kiawahensis* Tuomey and Holmes, 1856 as a likely synonym of *Tegula exoleta* (Conrad, 1843) following Dall [84] and Campbell [67]. *Agathistoma fasciatum* (Born, 1778) ranges across the extinction boundary.

### Turritellidae

Turritellidae are primarily semi-infaunal suspension feeders, also known to deposit-feed [85–87]. They have a diverse and abundant fossil record [87–89] and were disproportionately impacted by the PPWA extinction [2,3,5,6]. We excluded the turritellid genus *Vermicularia*, which has a different ecology (generally more reef-associated) and morphology (*Vermicularia* are uncoiled, making axial length comparisons non-analogous) when compared with other turritelld genera [90]. A recent phylogenetic treatment of Pliocene-to-recent species in Florida and the Atlantic Coastal Plain [66] indicated that at least three evolutionary lineages of non-*Vermicularia* turritelline gastropods were present, with two of these recognized as the genera *Caviturritella* and *Torcula*. Twenty fossil species were present in the Plio-Pleistocene of Florida and the Atlantic Coastal Plain, but only two remain in the region: *Torcula exoleta* (likely the direct descendant of *T*. *perattenuata* [66]) and *Turritella* (*sensu lato*) *perexilis* (= *Turritella acropora* [66]).

## Methods

Body size was represented by specimen length, recorded parallel to the coiling axis, and maximum body size for each species and mean sizes of fossil and recent specimens were investigated. Data were collected at the species level as differences in how species are assigned to genera may obscure real changes in community composition [91]. Cone snail species were evaluated using both maximum size and the typical size metric of Kohn [74]. Species synonymies and taxonomic assignments generally follow Hendricks [69], Friend et al. [66], and WoRMS [92]. An exception is that Hendricks [69] previously considered *Conasprella stearnsii* and *Conasprella jaspidea* synonymous, but Kohn [74] demonstrated that they are distinct; new measurement data were collected for these two species for this study with updated species determinations. The tegulid synonymy used herein is outlined in S2 Table. Newly collected specimen data were taken from specimens from the US Atlantic Coastal Plain or the Florida-Bahama Platform, however previously published data (e.g., data from Hendricks [69] and Kohn [74]), may have included specimens from outside of the present study region for wide-ranging species. Additional information on the methods used to determine each species’ body-size measurements are available in the supplemental text (S1 Text). Species measurement data used in our analyses are available in S3 Table along with species authority information.

When comparing the average size of species within each clade in the region before and after the extinction, we used the mean size of all species present in the region at the time to make the comparisons (whether each constituent species’ size was represented by the typical, average, or maximum size known for that specific species). We do not believe that “average size” is a real, emergent property that is held intrinsically by a higher taxon (clade), especially as the taxonomic assignment of a natural group of species to a certain rank is ultimately a human construct [91]. We are using these metrics as descriptors for how similarly conceptualized clades differ in aggregate properties in particular local instantiations at different time periods. A shift in the average body size for a given clade could be accomplished in a number of ways (e.g., preferential extinction, or speciation, of members which are smaller, or larger, than the original mean of the group, or greater changes during speciation towards smaller, or larger, body sizes). This descriptive property of a clade can however indicate whether macroevolutionary processes are favoring larger or smaller forms following the extinction event. We chose mean size rather than median as a size descriptor for our clades as they tended to be normally distributed and means were more likely to be impacted by the presence or absence of large or small outlier species, which could facilitate detection of changes that impacted which extremes were supported before or after the extinction.

Data were visualized and t-tests were performed in PAST v. 2.17c [93]. Additional statistical analyses (described below) were performed in R v. 4.3.2 [94] implemented in RStudio [95]. In Florida the extinctions may have occurred in two pulses [96], with the primary pulse at the Tamiami-Caloosahatchee boundary and a second pulse at the end of Caloosahatchee time. For our analyses we compared recent species with a pre-extinction fauna which included both Pliocene and earliest Pleistocene strata (Pinecrest beds of the Tamiami Fm., Caloosahatchee Fm., Nashua, Bear Bluff, and Chowan River Formations; see S1 Fig).

Using R [94], each family was evaluated using body-size data for all constituent species occurring in the region to determine whether the mean sizes of the recent species in the clade are aberrant relative to the size distribution of fossil species’ means for each family. Log-transformed data were used to evaluate body-size differences in each clade across the extinction, following the treatment of other multi-taxon datasets [97,98]. A size distribution for each family was generated by random resampling with replacement 1,000 times of N_m_ species’ log-transformed body sizes from among the pre-extinction interval fossil species, where N_m_ is the number of surviving species. This was used as the test distribution representing what we might expect if speciation or extinction were not biased towards or against large or small body sizes but were of equal magnitude to the total change in diversity, i.e. a “non-selective turnover.” This process was then repeated drawing N_f_, the number of fossil species from the pre-extinction log body-size distribution, and N_m_ from the modern log body-size distribution. We then compared the modern resampled distributions to the fossil and test distributions to determine whether extant species are larger or smaller than chance. If the mean of the modern resampled distribution fell within the 50% confidence window for test distribution, we concluded the turnover event did not alter the size distribution more than expected by chance. If the modern size distribution of a clade fell outside of this window, we concluded that the turnover event may have altered the average body size in the clade. This analysis was first performed using mean (Tegulidae and Turritellidae) or typical size (Conidae) for each species and then repeated using maximum size to represent each species’ size. For ancestor-descendant comparisons, data were visualized for each species in box plots, and 2-sample t-tests were conducted comparing ancestor-descendant populations.

## Results

### Clade-level analyses

**Fig 2:**
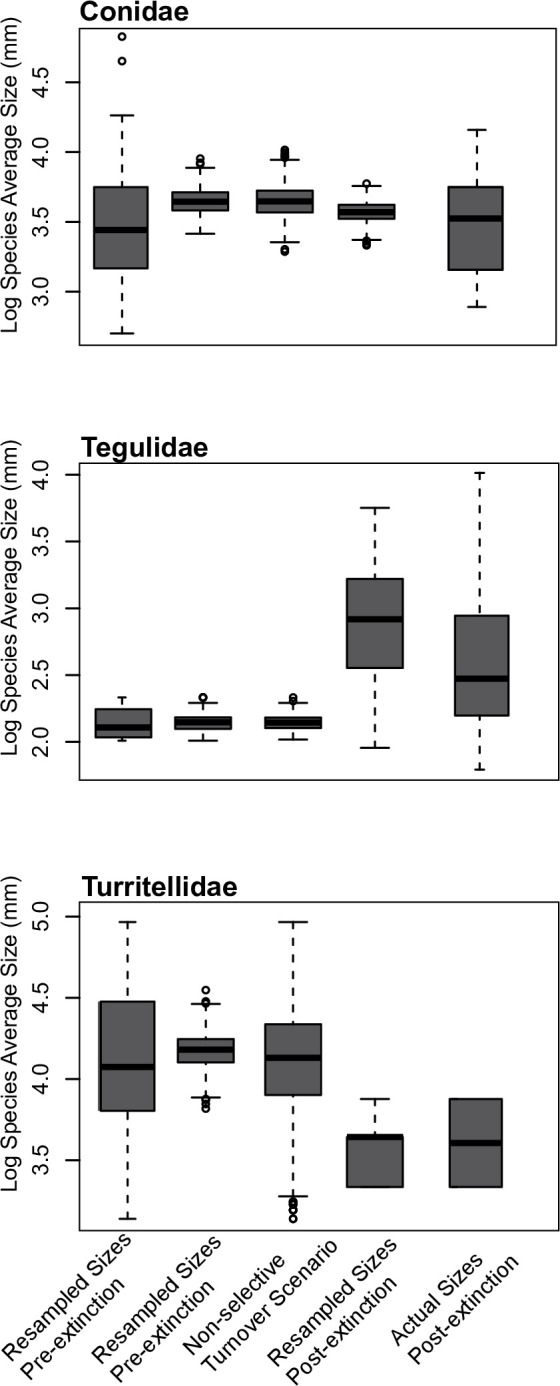
Clade body sizes using log transformed mean adult size to represent each turritellid and tegulid species’ size, and typical size to represent the size of each species belonging to Conidae (see Kohn [74] and supplemental text (S1 Text) for additional information on this methodology). Data are presented for each family from left to right, as the actual distribution of species body size prior to the extinction, a distribution of means when the data are resampled up to the number of pre-extinction species 1000 times with replacement, a non-selective turnover scenario represented by the distribution of mean sizes when the pre-extinction species’ sizes are resampled 1000 times using the number of extant species, a distribution of means when the modern species’ sizes are resampled 1000 times with replacement using the modern number of species for each sample, and the actual size distribution of the modern taxa.

For Conidae, the average species size was 40.9 mm (typical sizes) or 59.0 mm (maximum sizes) in the Plio-Pleistocene and 35.4 mm (typical sizes) or 62.0 mm (maximum sizes) in the modern, and these differences were not statistically significant (t>0.75, p = 0.44; t>0.19, p = 0.84 for typical and maximum sizes, respectively). Extinct species also did not differ significantly in size from surviving species (i.e., without considering species which originated post-extinction) by either metric. The mean of the resampled modern body-size distributions fell within the 50% confidence window of the non-selective turnover scenario, indicating no meaningful change (Fig 2). These results were replicated when maximum sizes were used to represent species’ sizes (S2 Fig).

**S2 Fig** Clade body sizes using log transformed maximum recorded size to represent each species’ size. Data are presented for each family, from left to right, as the actual distribution of species body size prior to the extinction, a distribution of means when the data are resampled up to the number of pre-extinction species 1000 times with replacement, a non-selective turnover scenario represented by the distribution of mean sizes when the pre-extinction species’ sizes are resampled 1000 times using the number of extant species, a distribution of means when the modern species’ sizes are resampled 1000 times with replacement using the modern number of species for each sample, and the actual size distribution of the modern taxa.

For Tegulidae, the average species size was 8.6 mm (mean sizes) or 11.5 mm (maximum sizes) in the Plio-Pleistocene and 19.0 mm (mean sizes) or 35.0 mm (maximum sizes) in the modern, but these differences were not statistically significant (for mean sizes, t = 1.1, p = 0.30, unequal variance t = 1.4, p = 0.23; for maximum sizes t = 1.01, p = 0.34, unequal variance t = 1.27, p = 0.26). The mean size of modern tegulids is heavily influenced by *Cittarium pica*, a very large species that originated in the late Pleistocene and which actually may not properly belong to Tegulidae (though it is closely related if not a member of the family). Excluding *Cittarium pica*, modern tegulids are 11.7 mm (the mean of each species average size) or 16.6 mm (the mean of each species maximum size), which preserves the direction of the signal (modern species are larger on average than Plio-Pleistocene species), but this difference is not statistically significant even when *C*. *pica* is included. Only one species survived the extinction, but it does not differ substantially in size from the species which became extinct (8.6 mm for surviving species versus 8.5 mm average size of extinct species). Resampled post-extinction means are substantially higher than for the non-selective turnover scenario, suggesting the modern tegulid species are larger than expected by chance (Fig 2). This result is replicated when maximum sizes are used to represent species’ body size (S2 Fig).

For Turritellidae, the average species size was 65 mm (mean sizes) or 96 mm (max sizes) for Plio-Pleistocene species and 38 mm (mean sizes) or 56 mm (max sizes) for the modern. These changes were not significant under a permutation t-test (t = 1.72, p = 0.10; t = 1.56, p = 0.14 for mean and maximum sizes, respectively), however only two turritellid species live in the region in the modern. Extinct species did not differ significantly in size from survivors (66 mm vs 62 mm—note that there was evolution, discussed below, in the *Torcula perattenuata*-*exoleta* lineage, and this comparison was made using the Plio-Pleistocene body-size distribution for this lineage). The resampled size distribution for the modern turritellids fell below the 50% confidence window of the non-selective turnover scenario, indicating that turritellids are smaller than expected by chance (Fig 2). When maximum sizes are used, this change is much less dramatic (S2 Fig). In this case, the actual mean falls within the central quartiles of the non-selective turnover scenario, although the resampled mean fell outside this window.

### Conidae species-lineage analyses

**Fig 3:**
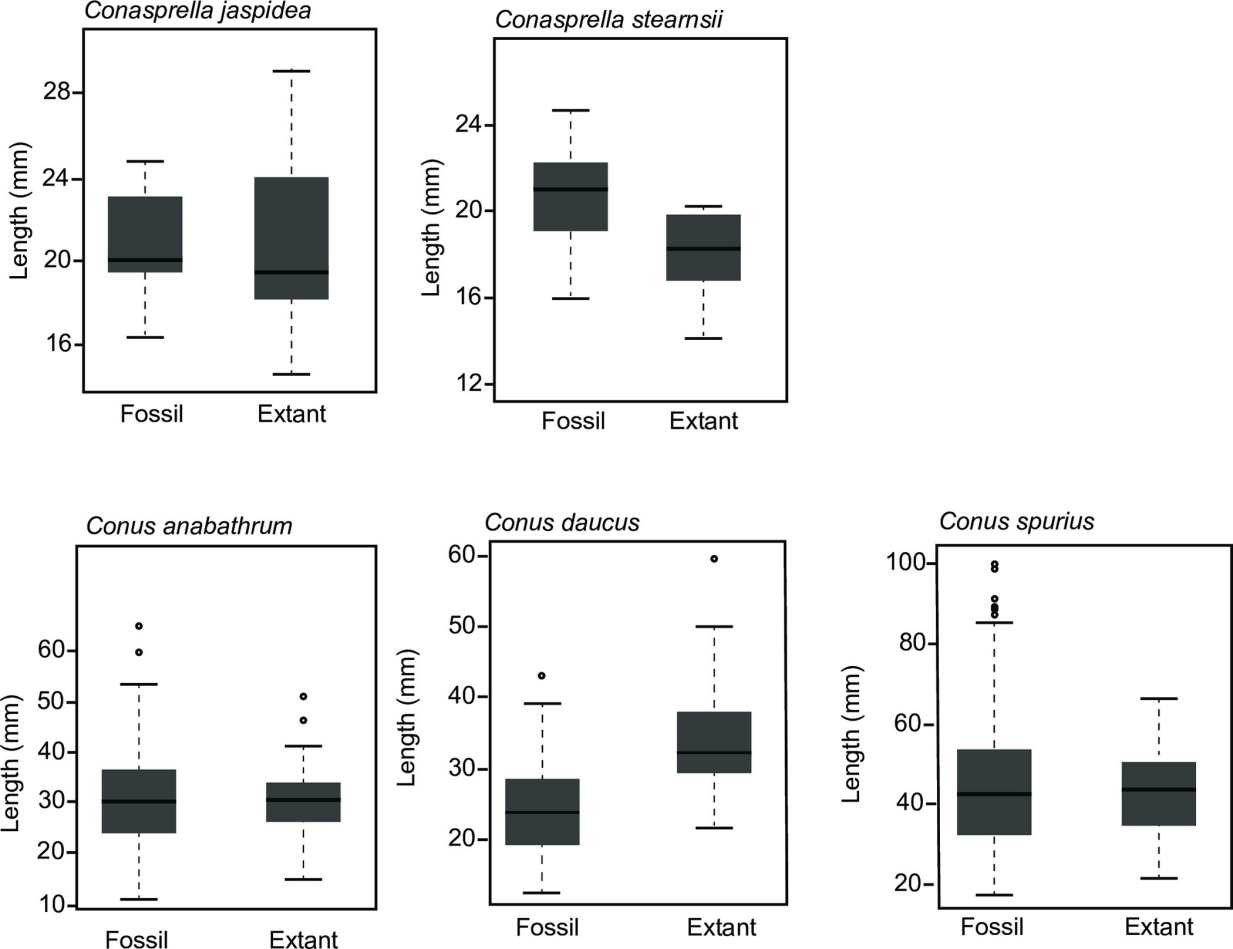
Shell-length distribution of species belonging to the family Conidae that survived the extinction event comparing Plio-Pleistocene to modern shells. Only *Conasprella stearnsii* and *Conus daucus* had statistically significant changes in body size.

Fossil and modern populations of *Conasprella jaspidea* (Gmelin, 1791), *Conasprella stearnsii* (Conrad, 1869), *Conus anabathrum* Crosse, 1865, *Conus daucus* Hwass, 1792, and *Conus spurius* Gmelin, 1791 were evaluated (Fig 3). One *Conasprella* species showed a statistically significant changes in body size between fossil and extant populations—modern *C*. *stearnsii* are smaller than their fossil counterparts (17.9 mm vs. 20.5 mm; t-test p <0.01), although this result was not significant when a Bonferroni [99] corrected α of 0.007 is applied). In contrast, one *Conus* species also showed a statistically significant difference, but of opposite sign: modern *Conus daucus* are larger than their fossil population (24.9 mm vs 34.5 mm; t-test p <0.001), a result which remains significant when using a Bonferroni corrected α of 0.007. *Conasprella jaspidea* did not change in size (20.8 mm for modern examples and 20.8 mm for fossil specimens; t-test p = 0.99). Excluding the measurements taken from figured specimens rather than measurements made directly from collections materials (7 specimens of *C*. *jaspidea* as detailed in S3 Table), the modern examples are smaller (18.3 mm) on average, but this difference is not statistically significant. Neither of the remaining *Conus* species showed statistically significant differences in specimen length in fossil versus modern populations. Extant *C*. *anabathrum* averaged 30.6 mm, while fossil *C*. *anabathrum* averaged 31.5 mm. Extant *C*. *spurius* averaged 42.1 mm in length, while fossil *C*. *spurius* averaged 45.4 mm (S3 Table).

### Tegulidae species-lineage analysis

**Fig 4:**
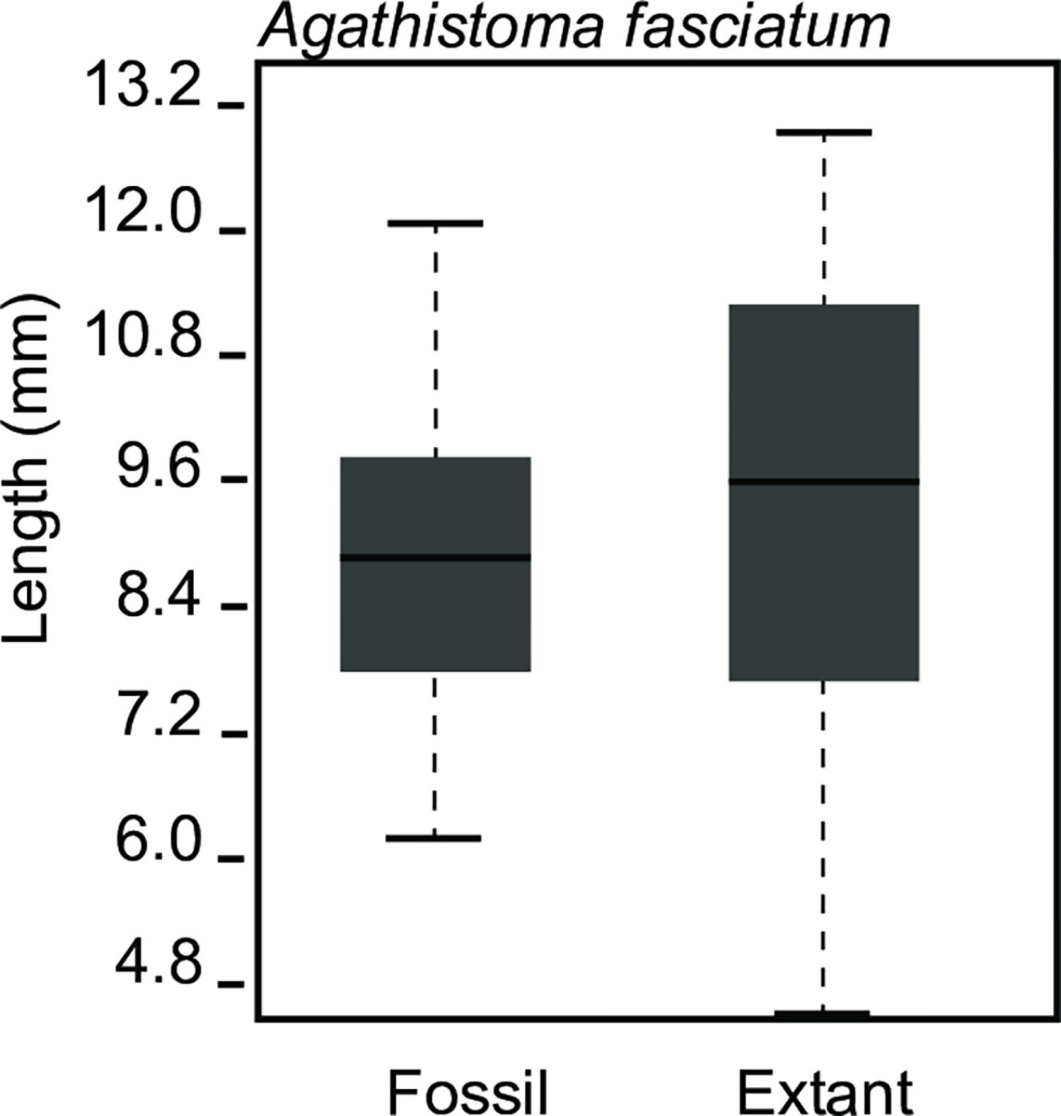
Shell length distribution for fossil and modern *Agathistoma fasciatum* comparing Plio-Pleistocene to extant shells. Modern *A*. *fasciatum* are not statistically distinct from fossil forms.

The single lineage which survived from the Pliocene, *Agathistoma fasciatum*, had an average fossil length of 8.6 mm and an average modern length of 9.4 mm, but this difference was not statistically significant (Fig 4).

### Turritellidae species-lineage analysis

**Fig 5:**
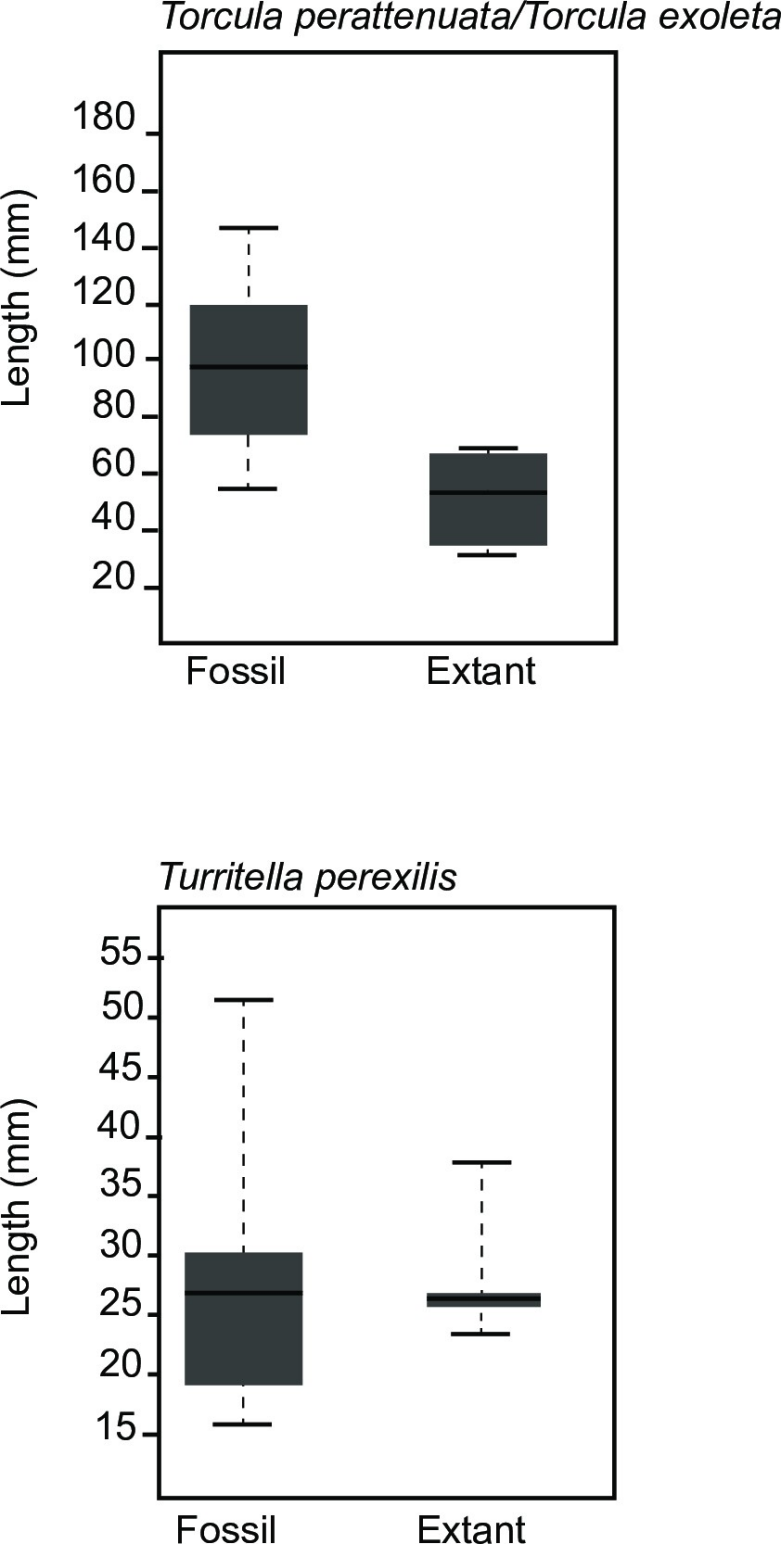
Shell length distribution of species belonging to the family Turritellidae which survived the extinction event comparing Plio-Pleistocene to extant shells. *Torcula perattenuata* is compared with *Torcula exoleta*, which is believed to be its anagenetic descendant following the phylogeny of Friend et al. [66] *Turritella perexilis* is the senior synonym of *Turritella acropora*, the name typically applied to extant forms [66].

One turritellid species-lineage, *Turritella perexilis* (= *T*. *acropora*) maintained a highly similar shell length across the turnover event (27.4 mm vs 28.1 mm for fossil and extant, respectively, a difference which was not statistically significant) (Fig 5). *Torcula exoleta* is, however, much smaller than its likely anagenetic ancestor *Torcula perattenuatta* [66], and this difference is statistically significant even after applying a Bonferroni correction (mean 48.3 mm vs 97.0 mm; t-test p <0.001; Fig 5).

## Discussion

### Body-size evolution and the meaning of trait changes in clades

Changes in body size among the species of molluscs in a region through time could be the result of: 1, natural selection on organismic properties; 2, ecological and environmental changes affecting phenotype (in the case of gastropods, temperature, availability of nutrients, and carbonate saturation may each have ecophenotypic effects); 3, macroevolutionary processes causing differential speciation and extinction rates between species with large body size versus small body size either directly or indirectly (if, for example, body size is linked to a species-level trait like geographic range size; or to another organismic trait such as larval type, that in turn is related to a species-level trait like geographic range [44,100–102], leading to substantial diversity differences [38]; 4, phylogenetic biases, if for some reason species with large-bodied organisms were more likely to give rise to species with large-bodied organisms, whereas species with small-bodied organisms were equally likely to give rise to small or large-bodied organisms [37]; 5, chance; and 6, a combination of some or all of these factors (see also Pietsch et al. [41]). In addition, once extinction eliminates a clade in a region, recovery in that group might be precluded, unless there were opportunities for biogeographic invasion, but these seem to have been limited in this case.

We may draw macroevolutionary rather than macroecological conclusions as the taxa considered herein, as well as the modern molluscan fauna in the region generally, are most likely the result of *in situ* speciation. The modern faunas of the US Atlantic Coastal Plain and Florida (the Caloosahatchian biogeographic province of Petuch [103]) are most similar to species that inhabited the region in the Pliocene (as the modern Caribbean molluscan fauna is most similar to the Pliocene Gatunian biogeographic province of Petuch [103]) indicating that the shift in taxonomic composition is most likely due to differences in *in situ* speciation rather than long-range dispersal [104,105]. Additionally, the life history traits of the taxa under study make it most likely that any Pleistocene-recent originations occurred locally. Extant tegulids have non-planktotrophic larvae [106–109] and most Conidae [74] and Turritellidae [17] in the modern western Atlantic have larvae expected to require little to no planktotrophy to metamorphose [74,110–112].

### How each clade behaved across the turnover event

The return to past levels of diversity of Conidae in Florida and the US Atlantic Coastal Plain included a return to a similar size distribution relative to the pre-extinction community; the very largest species were lost and new species have not yet achieved these large sizes. The impression that taxa are smaller may be the result of the “no-Gullivers” phenomenon [113], rather than a decline in species average body size following the extinction (either within lineages, the lilliput effect *sensu* Urbanek [114], or within a clade; see Abbott et al. [115] for further discussion of various uses of the term “lilliput effect” in the literature). In this case, we—as paleontological observers—are taking exceptional notice of the absence of one or two very large species, although the clade has a similar size distribution in the modern as it did prior to the extinction (see Harzhauser et al. [116] for a contrasting example where loss of large species was associated with overall decline in clade body size).

The evolution of larger species in Tegulidae may reflect a response to availability of the specific resources used by these species (seagrasses and algae growing on hard substrates [2,6,117]), despite overall decline in nutrient levels. Low connectivity [107] related to short duration in the plankton and high microhabitat partitioning [118] may both have contributed to high speciation and extinction (volatility) in this clade (although the relationship between larval mode and speciation rate in gastropods is not straightforward [102]).

Florida Tegulidae and Conidae originations appear to primarily occur post-extinction, with many recent species having no fossil record (Fig 1). Some extant species of Conidae may have Gelasian or Calabrian origins, but the rate of origination does not appear elevated (using the stratigraphic range data of Hendricks [69]). These clades that rediversified appear to have reoccupied much of the ecological space that may have been associated with their prior body-size distribution, especially in Florida where temperatures are similar to pre-extinction conditions. These new taxa may represent diversification within Florida and the US Atlantic Coastal Plain from the surviving species, immigration from neighboring regions, or in-situ radiation(s) following immigration events. Additional molecular genetic and phylogenetic work incorporating both fossil species and biogeographic information will be needed to determine precisely which species are of local origin. This post-extinction diversification appears to have different timing than originations in the southwest Caribbean where increased origination preceded extinctions [6,9].

Non-*Vermicularia* Turritellidae likely declined in size as a clade because the extinction was not followed by any cladogenesis in the surviving regularly-coiled taxa (*Caviturritella*, *Torcula* or *Turritella* s.l.), and because the genus *Caviturritella*, which was generally large bodied [119], was extirpated [66]. The extant regularly coiled western Atlantic Turritellidae may therefore be “dead clades walking” [17,120–122]. The decline of filter-feeding taxa associated with soft substrates is a pervasive characteristic of the turnover event [14], noted not only in turritellids but across bivalve taxa with similar ecology, including declines in the abundance of suspension-feeding bivalves compared to other feeding modes [6]. In contrast, cladogenesis continued to occur in *Vermicularia*, which differ from other turritellids in that many species are adapted to live on hardground environments, including reefs [90].

Gastropod taxa had higher speciation and extinction rates than bivalves across the turnover event [6] and, based on the patterns observed in our data, post-extinction speciation during recovery was an important part of body-size evolution and how this differed among gastropod families. The relatively rapid origination of many taxa which re-occupied a similar suite of body sizes to the extinct taxa also suggests that body size may be a particularly labile trait in gastropods. Bivalves have been documented to have decreased growth rates yet obtained similar sizes under the new environmental conditions by gaining longer lifespans [28,123] (see Palmer et al. [124] for an example from this study system). It is unknown whether gastropods exhibit similar patterns, but there is some evidence to the contrary in Turritellidae, which do not seem to have a higher frequency of long-lifespan species at high latitudes [58].

### Comparison to basal metabolic rates in PPWA molluscs

Recently Strotz et al. [1] found that bivalves and gastropod species containing organisms that had higher basal metabolic rates (BMRs)–which are associated with larger body sizes–had higher rates of extinction within the same study region and interval. The pattern was most pronounced in species with narrow geographic distributions and was more significant in bivalve species than gastropods. Because Strotz et al. [1] considered taxa from a broader range of taxonomic groups, used somewhat different taxonomic concepts and body-size data, and did not consider body size directly, but rather BMR, it is not possible to directly compare their results with ours. However, the difference in pattern may be attributable to several factors, including differences in number of species, differences in number of higher taxa, or relative proportions of taxa with narrow versus broad geographic range. Evaluating a broader suite of taxa and including additional data on functional traits, including larval mode, metabolic rate, lifespan, relationship to substrate, as well as information on species’ geographic range may help determine what selectivity occurred during the extinction, and better characterize how post-recovery communities differ from the Pliocene community.

## Conclusions

Gastropods are diverse in both species richness and ecological disparity. This ecological diversity complicates the identification of general rules for how taxa will respond to environmental change. The patterns of clade body-size evolution considered here did not directly correspond to microevolutionary changes within constituent lineages, except for the Turritellidae, the most severely impacted clade evaluated. More consistent patterns have also been recovered in analyses of bivalves than gastropods [1]. The turritellids are ecologically similar to most bivalves as semi-infaunal suspension and deposit feeders [87,88], suggesting that ecological factors were more significant for determining extinction impact than class membership. A weaker signal observed for selectivity in BMR in Gastropoda compared to Bivalvia [1] may therefore reflect the aggregation of a variety of gastropod groups which each responded in a manner consistent with their specific ecologies.

While there is a strong desire to find general rules which can be used to predict the ecological responses of taxa to environmental change, evaluations of maximally inclusive groups (e.g. benthic poikilotherms, mollusks, gastropods) must be balanced with studies of the natural history, ecology, and evolutionary responses of at least some of their constituent taxa or we may elide discovery of significant ecological-evolutionary relationships. Body-size evolution is an important potential evolutionary response to changing environmental conditions beyond extinction/migration, and our data suggest that the evolutionary response in this trait to the PPWA mass extinction was not generalizable across Gastropoda. Extrapolating from previously published data on mollusks generally across the PPWA event (including the bivalve case studies which have previously been important for characterizing the event’s impact) we might predict that decreased nutrient supply would have resulted in smaller body sizes for gastropods as well. However, in evaluating an ecologically diverse set of families of gastropods we found no class-level rule for gastropods, with each family behaving differently across the event (one increasing in size, one decreasing in size, and one remaining nearly identical in size distribution). Ecological differences, the impact of contingency in the form of extirpation or subclade extinction, and differences in recovery all appear to have contributed to the different responses we observed among gastropod families. Natural history data on a wider variety of taxa [125] and analyses evaluating the relationship between extinction, speciation, and ecological traits including body size [126,127] are urgently needed to assess what information is needed to best predict evolutionary responses to climate and other environmental changes.

## Supporting information

S1 Text Additional details of methods used to obtain size information and summary of supplemental files. (DOCX)

S1 Fig Regional stratigraphic correlation. (TIF)

S2 Fig Statistical analyses of clade body size across the event using maximum size to define each species’ body size. (TIF)

S1 Table Species assigned toe each time bin for text Fig 1. (XLSX)

S2 Table Tegulid synonymy used herein. (DOCX)

S3 Table All measurement data obtained for this study. (XLSX)
