# Supplementary material for: Body-size evolution in gastropods across the Plio-Pleistocene extinction in the western Atlantic
Source: PLoS One. 2024 Dec 13;19(12):e0313060. doi: 10.1371/journal.pone.0313060 (PMC11642969; doi:10.1371/journal.pone.0313060)
Supplement: S1 Text — (DOCX) [file pone.0313060.s001.docx]

**Supplemental Text 1:**

1. **Supplemental Methods**
2. **Figure S1.** Stratigraphic correlation of important Pliocene-Pleistocene fossiliferous units of Florida and the Atlantic Coastal Plain. Modified from Friend et al.,^66^ text fig 1.
3. **Figure S2.** Clade body-sizes using log transformed maximum recorded size to represent each species’ size. For each family data are presented, from left to right, as the actual distribution of species body size prior to the extinction, a distribution of means when the data are resampled up to the number of pre-extinction species 1000 times with replacement, a non-selective turnover scenario represented by the distribution of mean sizes when the pre-extinction species’ sizes are resampled 1000 times using the number of extant species, a distribution of means when the modern species’ sizes are resampled 1000 times with replacement using the modern number of species for each sample, and the actual size distribution of the modern taxa.
4. **Table S1.** Diversity and species stratigraphic occurrences.
5. **Table S2.** Synonymy used for Tegulidae in this manuscript.
6. **Table S3.** Body Size measurement Data.

**Supplemental Methods**

**Sources of Body size data**

Data on taxon body-size was compiled from data collected in support of monographic treatments of Florida fossil Turritellidae (Friend et al., 2023), Conidae (Hendricks, 2009), and targeted examination of additional museum specimens held at the Paleontological Research Institution (PRI) in Ithaca, NY, USA and the Florida Museum of Natural History (UF) in Gainesville, FL, USA. All body size data collected for this study to supplement Friend et al. (2023) and Hendricks (2009) was collected using specimens from the Florida-Bahama platform. Where specimen body size data were obtained from the literature, the associated specimens were reposited at the American Museum of Natural History (AMNH) in New York, NY, USA, the Smithsonian Institution (USNMIP) in Washington, D.C., USA, and the Harvard Museum of Comparative Zoology (MCZ) in Cambridge, MA, USA.

The most comprehensive recent treatment of modern western Atlantic Conidae which reported body-size information for extant species (Kohn, 2014) reported species size in terms of both maximum length and typical size. We utilized these data to characterize modern maximum and typical body-size for extant species, although specimens outside of our study region contributed to Kohn’s (2014) body-size estimates for species with broad geographic ranges. Typical size was defined as the median of all shell lengths when those which were below half of the maximum reported shell length were excluded (removing, for example, juvenile specimens), a technique attributed to U. Smith (Kohn, 2014). Fossil measurement data collected from Hendricks (2009) were treated in the same manner to develop typical size calculations for Conidae which could be compared between fossil and modern species and between fossil and modern representatives of extant species with a fossil record. Following the taxonomy of Kohn (2014), new data were collected from the collections at PRI for *Conasprella stearnsii* and *Conasprella jaspidea* as these were considered synonymous in Hendricks (2009). Definite examples of *Conasprella jaspidea* were less well represented in PRI collections (fossil N=8, modern N=7), but figured specimens of Kohn (2014) were available to increase the size of the modern dataset. In total 8 *C. jaspidea* and 22 *C. stearnsii* from the Plio-Pleistocene of Florida which met the typical size cutoff for each species were measured. The largest confirmed fossil *C. stearnsii* found (PRI 53184; 28.4 mm) was from the Pleistocene Bermont Fm, with the largest pre-extinction specimen (PRI 54410; 24.7 mm) being found in the Pinecrest Beds.

Data for Tegulidae and Turritellidae which have not previously published are based on materials in the collection of the PRI, except for Pleistocene fossils of *Cittarium pica*. Fossil specimens of *Cittarium pica* were not available at the PRI and therefore specimens housed at UF were also included for this species. Additionally, literature references were used to determine maximum reported sizes in the region, and average sizes for specimens that were rare or absent from our collections. The overwhelming majority of modern Tegulid specimens in PRI collections belonged to *Agathistoma fasciata* or *Cittarium pica*, although several other Tegulids occur in the region according to GBIF data (GBIF.org (30 July 2023) GBIF Occurrence Download [https://doi.org/10.15468/dl.eks99a](about:blank)). We therefore examined literature data to determine average and maximum reported body-sizes for the remaining species (Tryon, 1889; Warmke and Abbott, 1961; Humfrey, 1975; Rehder, 1981; Robertson, 2003; Dornellas et al., 2022; Ahyong et al., 2023). It is notable that *Cittarium pica* are fished for human consumption, second in exploitation to the queen conch *Aliger gigas* (*Strombus gigas*), and are consequently well studied in the modern (Randall, 1964; Cervigon et al., 1993; Schmidt et al. 2002; Robertson, 2003). High human exploitation results in both decreased density and smaller specimen sizes for this species (Schmidt et al., 2002; Robertson, 2003), as human exploitation frequently disproportionately impacts large individuals (Robertson, 2003; Bosch et al., 2022; Huang et al. 2023). Large shells are therefore unusual in areas of human exploitation with maximum sizes coming from reports of shells collected from isolated areas (See Robertson, 2003).

In Turritellidae, specimen lengths often needed to be reconstructed using the theoretical apex system of Johnson et al. (2017), which calculates missing apical length from the widths of the smallest and largest whorls and the distance between these measurements. To determine species sizes in a comparable way within this family, 10 specimens were selected to represent each turritellid species by randomly assigning a number to each lot. A random number generator was used to select a lot, and the largest specimen from that lot was measured. If a lot was redrawn, the next largest individual in the lot was then measured. When a lot consisting of a single specimen was randomly drawn repeatedly, that individual was resampled. If fewer than five lots were present, the largest of each was measured prior to resampling, counting as many different individuals larger than two whorls as possible. Specimens figured in Friend et al. (2023) were used to represent rare turritellid species not present in PRI collections.

**Supplementary References**

Bosch NE, Monk J, Goetze J, Wilson S, Babcock RC, Barrett N, Clough J, Currey‐Randall LM, Fairclough DV, Fisher R, Gibbons BA. Effects of human footprint and biophysical factors on the body‐size structure of fished marine species. Conservation Biology. 2022 Apr;36(2):e13807.

Cervigón F, Cipriani R, Fischer W, Garibaldi L, Hendrickx M, Lemus AJ, Márquez R, Poutiers JM, Robaina G, Rodriquez B. Field guide to the commercial marine and brackish-water resources of the northern coast of South America.

Huang S, Edie SM, Collins KS, Crouch NM, Roy K, Jablonski D. Diversity, distribution and intrinsic extinction vulnerability of exploited marine bivalves. Nature Communications. 2023 Aug 15;14(1):4639.

Humfrey, M. Sea Shells of the West Indies. Collins, London, 1975; 351 pp.

Johnson EH, Anderson BM, Allmon WD. What can we learn from all those pieces? Obtaining data on drilling predation from fragmented high-spired gastropod shells. Palaios. 2017 May 1;32(5):271-7.

Randall HA. A study of the growth and other aspects of the biology of the West Indian topshell, *Cittarium pica* (Linnaeus). Bulletin of Marine Science. 1964 Jul 1;14(3):424-43.

Rehder, HA, National Audubon Society Field Guide to North American Seashells. Knopf, New York, 1981; 894 pp.

Robertson R. The edible West Indian “whelk” *Cittarium pica* (Gastropoda: Trochidae): natural history with new observations. Proceedings of the Academy of Natural Sciences of Philadelphia. 2003 Dec;153(1):27-47.

Schmidt S, Wolff M, Vargas JA. Population ecology and fishery of *Cittarium pica* (Gastropoda: Trochidae) on the Caribbean coast of Costa Rica. Revista de biología tropical. 2002 Dec;50(3-4):1079-90.

Tryon GW. Manual of Conchology, structural and systematic: with illustrations of the species. Academy of Natural Sciences. 1889; 11; 1-519.

Warmke, GL, Abbott, RT. Caribbean Seashells: A Guide to the Marine Mollusks of Puerto Rico and other West Indian Islands, Bermuda and the Lower Florida Keys. Livingston Publishing Company, Narberth, Pennsylvania,1961; 348 pp.
