# Supplementary figures and images for: Body-size evolution in gastropods across the Plio-Pleistocene extinction in the western Atlantic
Source: PLoS One. 2024 Dec 13;19(12):e0313060. doi: 10.1371/journal.pone.0313060 (PMC11642969; doi:10.1371/journal.pone.0313060)

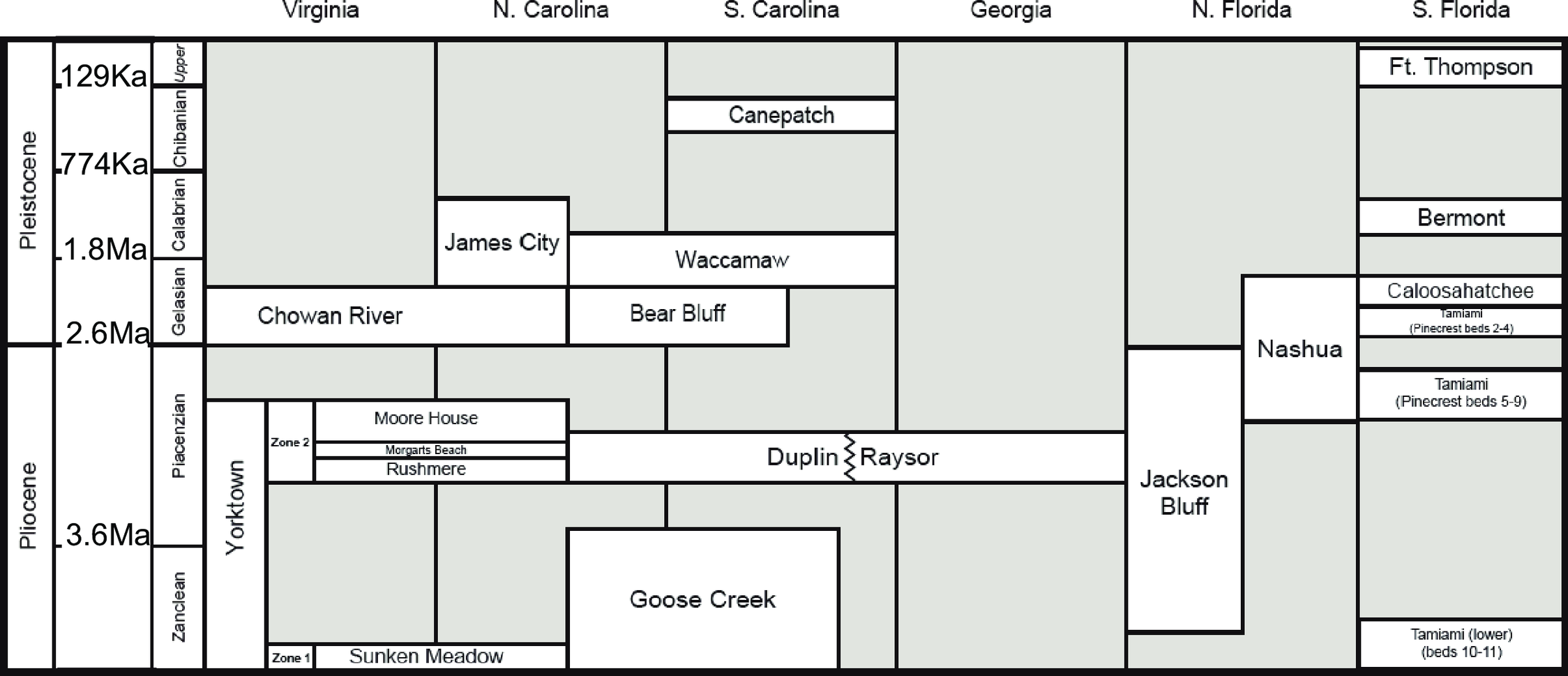

Supplement: S1 Fig — (TIF) [file pone.0313060.s002.tif]

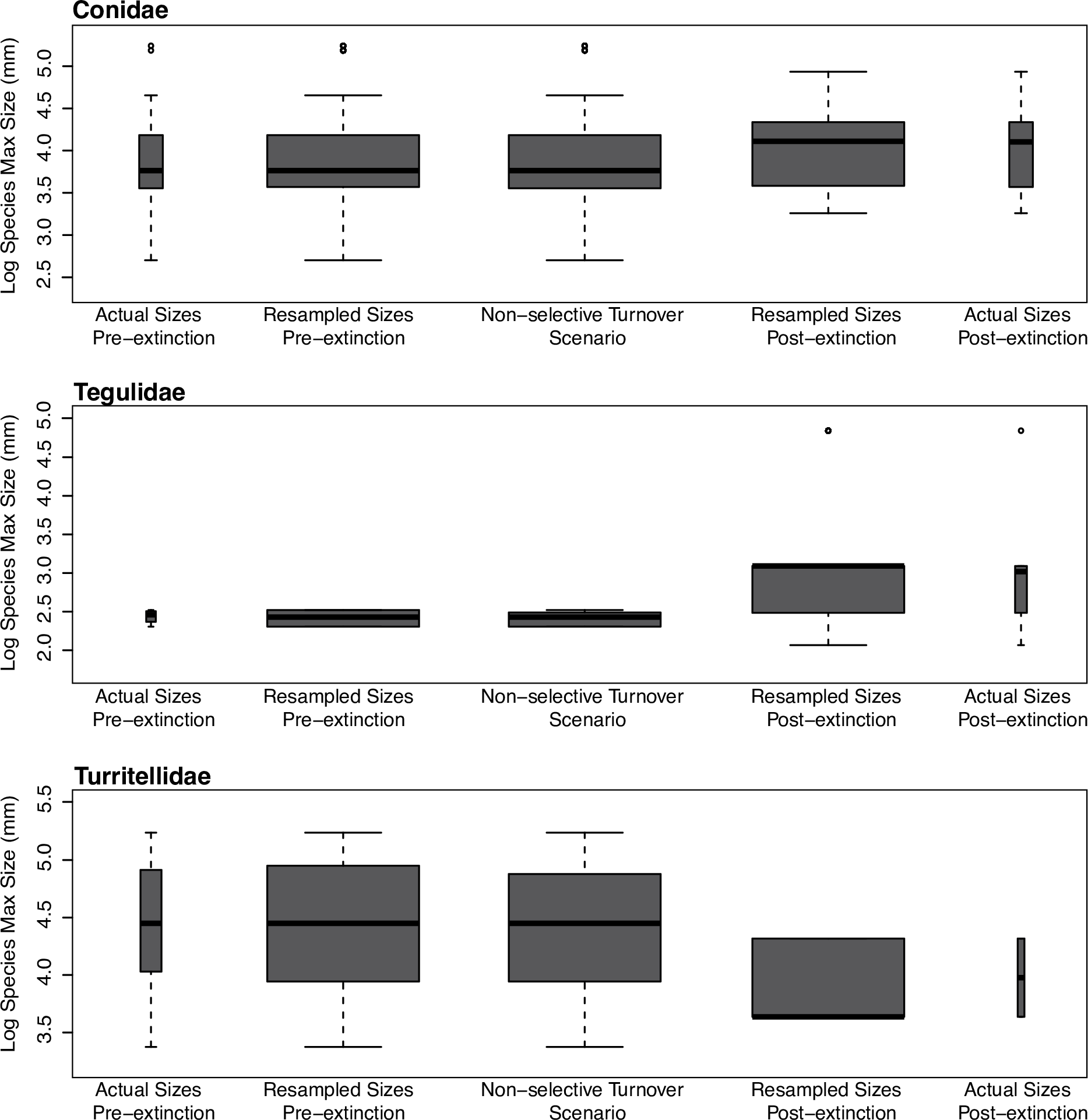

Supplement: S2 Fig — (TIF) [file pone.0313060.s003.tif]
