# Supplementary material for: Body-size evolution in gastropods across the Plio-Pleistocene extinction in the western Atlantic
Source: PLoS One. 2024 Dec 13;19(12):e0313060. doi: 10.1371/journal.pone.0313060 (PMC11642969; doi:10.1371/journal.pone.0313060)
Supplement: S2 Table — (DOCX) [file pone.0313060.s005.docx]

**Table S2**. Tegulidae synonymy used herein.

| **Family** | **Species** | **Authority** | **Synonyms** |  |
| --- | --- | --- | --- | --- |
| Tegulidae | *Agathistoma fasciatum* | (Born, 1778) | *Aderorbis picta*  *Chlrostoma fasciata,*  *Tegula fasciata,*  *Tegula fasciatum, Trochus fasciatus* | Teinison Woods, 1877  (Born, 1778)  (Born, 1778)  (Born, 1778)  Born, 1778 |
|  | *Agathistoma lividomaculatum* | (C.B. Adams, 1845) | *Astele turbinate, Chlrostoma scalaris,*  *Monilea turbinate, Monodonta lividomaculata, Tegula lividomaculata, Trochus sclaris* | (Tenison Woods, 1877)  (Philippi, 1844)  Tenison Woods, 1877  Adams, 1845  (Adams, 1845)  Philippi, 1844 |
|  | *Agathistoma hotessierianum* | (D’Orbigny, 1842) | *Monodonta maculostriata, Tegula hotessieriana, Tegula maculostriata, Trochus hotessierianus* | (Adams, 1845)  (d’Orbigny, 1842)  (Adams, 1845)  d’Orbigny, 1842 |
|  | *Cittarium pica* | (Linnaeus, 1758) | *Livona pica, Trochus picoides, Turbo pica* | (Linnaeus, 1758)  Gould, 1853  Linnaeus, 1758 |
|  | *Tegula gruneri* | (Phillippi, 1849) | *Trochus gruneri*  *Chlorostoma substriatum* | Philippi, 1849  Pilsbry, 1889 |
|  | *Tegula excavata* | (Lamarck, 1822) | *Trochus excavatus* | Lamarck, 1822 |
|  | *Tegula calusa*† | Petuch, 1994 |  |  |
|  | *Tegula exoleta*† | Conrad, 1843 | *Monodonta exoleta*  *Monodonta exoluta*  *Chlorostoma exoletum*  *Tegula kiowahensis* | (Conrad, 1843)  Conrad, 1843  Dall, 1892  (Tuomey and Homes, 1856) |
|  | *Tegula lindae*† | Petuch, 1994 |  |  |
